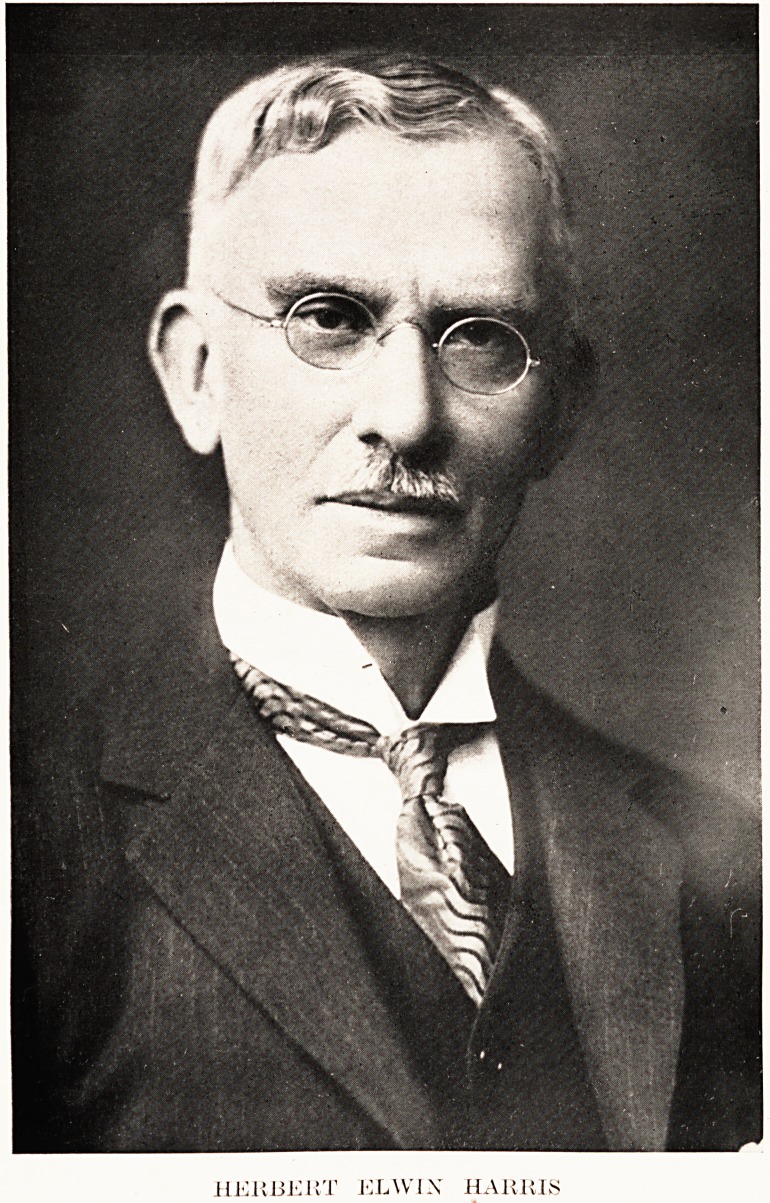# H. Elwin Harris

**Published:** 1941

**Authors:** 


HERBERT ELWIN HARRIS

### Obituary

### H. ELWIN HARRIS, M.A., M.B., F.R.C.S.

Herbert Elwin Harris, formerly of Bristol, who died on 20th May, was born in 1860 at Binham in Norfolk and was baptized in the beautiful old Norman abbey church there. His father, like many of his forbears, was a gentleman farmer who lived in a house facing the green; his mother, a Miss Wrench, came of another old Norfolk family, and through her, Dr. Elwin Harris was descended from Sir Thomas Browne, the author of *Religio Medici*.

He was educated at his uncle's school in Truro, whence he proceeded to Christ's College, Cambridge, and graduated in the Mathematical Tripos in 1882. He was destined for holy orders, but he persuaded his college tutor to allow him to read medicine as well as mathematics and he passed his first M.B. whilst preparing for the Tripos. After this his father consented to his continuing the medical curriculum and he went from Cambridge to St. Bartholomew's, where he was a dresser under Sir William Savory. He took the qualifications of M.R.C.S. in 1885, L.R.C.P. in 1886, and M.B. Cambridge in 1886. After leaving Bart's he was a house-surgeon at Plymouth and then worked for several years in various London poor-law infirmaries, ending with a long period at St. Saviour's, Dulwich. In 1896 he took the F.R.C.S. and settled in general practice in Bristol, where he was soon appointed anæsthetist at the Royal Hospital for Women and Children. A few years later he was elected to a vacancy as surgeon and became also surgeon in charge of the ear, nose, and throat department.

During the 1914-18 war Dr. Harris was captain R.A.M.C. (T.) on the *à la suite* staff of the Second Southern (Territorial) General Hospital and worked in the Beaufort War Hospital at Fishponds, Bristol. He was one of the last in Bristol to combine general practice with operative surgery, and he achieved success in both branches of practice. During his earlier years in practice Dr. Harris was almost hobbyless. His recreation was cycling, and in the fashion of the time he would ride vast distances in a day. Presently, however, he became an enthusiastic gardener and photographer. He was one of the first doctors in Bristol to drive a motor car, and he was distinguished by favouring an open two-seater with a blue body and yellow wheels, which always looked new. However often he changed his old car for a new one he retained the original registration number.

Dr. Harris was President of the Bristol Medico-Chirurgical Society in 1933-34, and for forty-two years was a member of the British Medical Association. In 1934 he retired from practice to live for the rest of his life at Halse, near Taunton. There he died suddenly of coronary thrombosis, in the garden he so dearly loved, within a few weeks of his eighty-first birthday. Dr. Harris was widely known and well loved, especially by his patients, in whose interests he worked himself unsparingly. He yielded to no one in his admiration for his old teacher, Sir James Paget, a Norfolk man like himself, from whom perhaps he had caught something of the kindly courtesy which was one of his outstanding characteristics. He was succeeded in his practice by his son, who had helped him for some ten years before his death.

To his son and his daughter we offer our deepest sympathy.

[Reprinted, by kind permission, from *The British Medical Journal*, 14th June, 1941.]

## Figures and Tables

**Figure f1:**